# The Complementary Roles for Plant-Source and Animal-Source Foods in Sustainable Healthy Diets

**DOI:** 10.3390/nu13103469

**Published:** 2021-09-29

**Authors:** Kevin B. Comerford, Gregory D. Miller, Wendy Reinhardt Kapsak, Katie A. Brown

**Affiliations:** 1 OMNI Nutrition Science, Davis, CA 95618, USA; 2 California Dairy Research Foundation, Davis, CA 95618, USA; 3 National Dairy Council, Rosemont, IL 60018, USA; gregory.miller@dairy.org (G.D.M.); katie.brown@dairy.org (K.A.B.); 4 Produce for Better Health Foundation, St. Louis, MO 63144, USA; wendy@pbhfoundation.org

**Keywords:** fruits, vegetables, dairy, plant-based, plant-source foods, animal-source foods, food groups, sustainability, food-based dietary guidelines

## Abstract

There are approximately 100 countries with food-based dietary guidelines throughout the world, each of which aims to encompass the cultural, geographical, and health considerations unique to their country of origin. Common themes throughout these guides emphasize diverse and balanced intake of food groups from both plant- and animal-sources. With the globally recognized need to shift to more sustainable food systems, several countries and international food and health organizations have begun to incorporate sustainability recommendations into their dietary guidance. These sustainability recommendations are often based on food source (i.e., eat more plant-source and fewer animal-source foods), yet food source may not be the only useful or informative comparator for assessing healthy and sustainable diets. The purpose of this narrative review is to examine the roles of plant-source foods and animal-source foods in the context of sustainable healthy diets—with an emphasis on the contributions of the most commonly recommended food groups from global food-based dietary guidelines (i.e., fruits, vegetables, and dairy foods). Overall, plant and animal agriculture have complementary and symbiotic roles in healthy and sustainable food systems, and these abilities are largely dependent on various contextual factors (e.g., geography, production practices, processing methods, consumption patterns)—not just on whether the food originated from the plant or animal kingdom.

## 1. Introduction

The primary objectives of food-based dietary guidelines (FBDGs) around the world are similar; to provide evidence-based food and nutrition guidance for various audiences (including policymakers, health professionals, educators, and/or consumers), for the purpose of fostering healthy eating habits and reducing non-communicable diseases among the general population [1,2]. At present, there are approximately 100 countries with national FBDGs throughout the world, most of which provide a food guide that aims to encompass the cultural, geographical, and health considerations unique to their country of origin [3]. The most prevalent dietary advice being promoted in FBDGs worldwide can be broken down into three key messages; (1) consume a variety of nutrient-rich foods from different food groups, (2) achieve balanced intake of fruits, vegetables, legumes, cereals, and animal-source foods (ASFs), and (3) limit intake of sugar, fat, and salt [1,2]. However, even within the most basic of these principles, there are several contextual factors and tradeoffs that deserve further consideration since dietary guidance can differ markedly depending on factors such as geography (both within and among countries), demographics (e.g., age, sex, life stage), and whether sustainability factors are considered or not. The purpose of this narrative review is to examine the roles of plant-source foods (PSFs) and ASFs in the context of global FBDGs and sustainable healthy diets—with an emphasis on the most commonly recommended food groups from FBDGs (i.e., fruits, vegetables, and dairy foods). To the best of our knowledge, this is the first examination in the literature comparing global FBDG messaging, dietary contributions, and sustainability impacts for these foods.

## 2. Dietary Components Provided by Plant-Source Foods and Animal-Source Foods

Despite coming from two separate taxonomic kingdoms of life, PSF and ASF contain an overlapping supply of nearly all macronutrients and micronutrients essential for human health—although, the quantities, qualities, ratios, and combinations of these nutrients may differ considerably between them. These differences are often exemplified with ASFs tending towards higher quantities and bioavailability of protein, branched-chain amino acids, iodine, iron, zinc, vitamin B12, choline, taurine, creatine, and carnosine [4,5,6]; and PSFs containing more carbohydrates, (starches, sugars, and fibers), vitamin C, and numerous plant secondary metabolites, which are recognized for their antioxidant, anti-inflammatory, and antimicrobial properties [1,7] (Table 1, Table 2 and Table 3). PSFs and ASFs also tend to differ in their essential fatty acid profiles and fat-soluble compounds, with ASFs generally containing more metabolically active forms of omega-3 fatty acids (i.e., EPA and DHA), and more bioavailable forms of vitamins A and D; and PSFs being more common sources of various unsaturated fatty acids and vitamin E (Table 1 and Table 2). In essence, PSFs and ASFs can each provide an array of dietary compounds that impact human health, many of which are present in both plant and animal sources, and many that are only present in one or the other (Table 1, Table 2 and Table 3).

## 3. Food-Based Dietary Guidance for Plant- and Animal-Source Foods

All global FBDGs encourage the consumption of PSFs (especially fruits and vegetables), and the majority encourage the consumption of ASFs (especially dairy foods) [1]. However, the specifics of these recommendations may differ considerably by region. For example, FBDGs from many low- and middle-income countries (LMICs) located in Africa, Asia and Latin America tend to emphasize the intake of various ASF in order to prevent nutrient deficiencies (especially for protein, vitamin A, calcium, iodine, iron, and zinc) [13,14] and to promote the proper growth and development of children [15,16]. In high-income countries (HICs) such as in the U.S. and several European nations, FBDGs tend to take a different approach, aimed at reducing chronic disease risk and emphasizing the intake of certain ASF over others (e.g., dairy, eggs, fish and poultry over red meat), and specific subtypes over others (e.g., low-fat and unprocessed options over more energy-dense and processed options) [1,14,17,18]. These diverging recommendations between regions highlight the unique differences in nutritional imbalances that occur across the globe, and emphasize the complexities involved in developing a single set of dietary guidelines that aim to inform the dietary patterns of an entire country’s population.

## 4. Food Group Recommendations and Key Messaging in Food-Based Dietary Guidelines

FBDGs provide guidance in terms of foods, food groups, and dietary patterns [19]. All FBDGs (or their related food guides) mention or depict food source (i.e., PSF and ASF), but the majority of their key messaging is based on food groups [1]. While most of these food groups are based on the food’s origin (i.e., from specific types or parts of plants and animals), such as fruits, vegetables, grains, legumes, nuts, dairy, and meat, some food groups are based on individual nutrients (i.e., “starchy staples” or “protein foods”) [1,20]. These nutrient-based classifications cut across food groups in the case of starch (e.g., rice; grain, potato; vegetable, or plantain; fruit), and across kingdoms in the case of protein (e.g., some FBDGs includes nuts, seeds, and legumes in the “protein foods” group) [1]. Overall, most FBDGs recommend the intake of two or more times the number of PSF groups (e.g., fruits, vegetables, grains, legumes, nuts, and starchy staples) as ASF groups (e.g., dairy, meat, protein foods). Most FBDGs also recommend the intake of a greater number of servings, and larger portions of PSFs than ASFs, making FBDG-recommended patterns throughout the world primarily plant-based dietary patterns [1].

For PSFs, key messaging specifically encouraging fruit and vegetable intake is included in more than 90% of FBDGs, far exceeding that of any other food group (starchy staples < 70%, legumes and nuts < 60%). Fruits and vegetables are also the only food group(s) with consistent quantitative messaging among FBDGs (~5 or more portions/day; or ~400 grams/day), but there is little consistency as to the specific number of servings, portion sizes, or total daily amounts recommended for the other food groups [1]. As for ASFs, key messaging regarding the consumption of milk and dairy foods exceeds that for any other ASF (dairy > 70%, fish < 60%, meat < 55%, eggs < 35%, poultry < 30%), with the caveat that a minority of countries also include messaging to limit or moderate the intake of certain types of ASFs such as processed meat, high-sodium cheese, and fish that could contain mercury [1]. Overall, approximately 70% of FBDGs recommend regular dairy intake, typically ranging between 1–4 servings/day depending on the region [14].

Overall, the fruit, vegetable, and dairy food groups, which represent dietary options from both the plant and animal kingdoms, are the most prevalently recommended food groups in global FBDGs [1]; yet these food groups are also chronically underconsumed throughout the world, and therefore deserve further attention for their roles in dietary patterns [21,22,23,24].

## 5. The Contributions of Fruits, Vegetables, and Dairy Foods to Diet Quality

Nearly all fruits and vegetables are considered to be nutrient-dense foods [25], and given their basic botanical similarities, fruits and vegetables are often treated similarly and/or classified together in dietary guidance [1]. However, fruits and vegetables are not at all nutritionally or phytochemically equivalent. In general, fruits are higher in fructose, vitamin C, and phenolic compounds such as flavonoids and proanthocyanidins, while vegetables tend to be higher in protein, starch, minerals, fat-soluble vitamins, and the bioactive compounds glucosinolates, phenolics, and carotenoids [11,26,27] (Table 1). The macronutrient content can also vary widely between fruits and vegetables with some being much higher in fat and lower in sugar (e.g., coconuts and olives), and others being higher in protein and complex carbohydrates (e.g., peas and potatoes). There are also considerable differences within the foods in each group (e.g., apples and avocados are both fruits with very different fat, fiber, micronutrient, and phytochemical profiles), however, these intra-group differences are generally not addressed in dietary guidance. Hence, the general guidance in FBDGs to consume a diversity of foods applies both across food groups (e.g., consume foods from all the different food groups), and within food groups (e.g., consume a diversity of different foods from each food group). The unique and potentially synergistic combinations of micronutrients, fiber, and bioactive phytochemicals found in diets that are rich in a variety of fruits and vegetables are universally associated with higher diet quality, reductions in the risk for several chronic diseases, and the promotion of overall health and wellbeing [11,26,28,29].

Unlike fruits and vegetables, which can be sourced from hundreds of different plants, dairy products come from one source—milk. However, there is still a huge diversity of dairy products to choose from as this milk may come from different types of animals and can be processed in dozens of different ways. Additionally, some FBDGs, primarily from North America, Europe and Asia, also classify calcium-fortified soy beverages, yogurts and/or cheeses as part of the dairy food group, resulting in hundreds of different dairy products that may meet FBDG recommendation criteria [14]. Similar to fruits and vegetables, FBDG recommended dairy foods, which are primarily different forms of milk, cheese, yogurt and kefir, can vary significantly in their protein, fat, vitamin, mineral, and bioactive components (especially probiotics) combinations and ratios. Regardless of their fat level, most dairy foods are nutrient-rich foods that contain greater amounts of U.S. DGA shortfall nutrients (calcium, potassium, and vitamin D) per serving than most any other food [17,30]. Due to the vast range of potentially beneficial dietary components found in dairy foods, consuming a diversity of these products (especially those which are fermented and/or fortified), is associated with several measures of diet quality [31,32,33], and beneficial health effects ranging from the prevention of multiple nutrient deficiencies to a reduced risk for colorectal cancer, metabolic syndrome, and type 2 diabetes [14,34].

## 6. Dietary Patterns and Health Outcomes—The Importance of Balance

Unfortunately, most countries’ consumption patterns do not align with their FBDGs’ recommendations, and these imbalances in food group and nutrient intake can differ considerably by region [35]. For example, countries in South East Asia such as Indonesia and Bangladesh manage to follow their FBDGs more closely than average, achieving several of their recommended consumption targets [35]. On the other hand, North American countries such as Canada and the U.S. achieve few to none of their FBDGs recommended consumption targets [35]. In the U.S., greater than 90% of the total population do not meet the recommended vegetable intake levels, and approximately 80% do not meet the recommendations for fruit or dairy intake [21,22]. The majority of the U.S. population is also simultaneously overconsuming foods and energy from the other food groups as well as from discretionary foods that are high in sugars and fats [10]. Likewise, much of the global population is also habitually eating out of balance with their FBDGs, with incidences disproportionately affecting LMICs [36]. Nearly three-fourths of the global population is regularly consuming fewer than the recommended amounts of fruits and vegetables [23] and a majority of the global population is consuming significantly less dairy foods than recommended [24]. 

Unbalanced dietary patterns that are low in the most highly recommended food groups (fruits, vegetables, and dairy foods) are associated with higher rates of obesity, chronic disease, mortality, and morbidity [17]. These negative health effects are exacerbated when FBDG recommended foods/food groups are replaced with options that are nutrient-poor and contain excessive quantities of sugar, fats, and salt - making it difficult to achieve nutrient adequacy without overconsuming energy [17]. The challenge of achieving a healthy dietary pattern, which is both balanced in nutrient and energy intake, puts a premium on affordable and acceptable foods that are both nutrient-dense and good sources of one or more commonly underconsumed nutrients.

At the global level, the most commonly underconsumed nutrients are calcium, iron, zinc and vitamin A [37]; which are all primarily found in higher quantities and more bioavailable forms in ASFs; while in the U.S. the most commonly underconsumed nutrients are: fiber, potassium, magnesium, calcium, iron, zinc, vitamin A, vitamin C, vitamin D, and vitamin E [17,30]. No single food source or group contains adequate amounts or ideal ratios of all of these nutrients. Rather they are spread out among the food groups (Table 1). Therefore, the majority of FBDGs emphasize the consumption of a complementary combination of PSF and ASF groups for achieving nutrient adequacy and overall healthy dietary patterns. It is important to note that adequate nutrient intake is not synonymous with a healthy dietary pattern, especially if the dietary pattern contains excessive levels of nutrients and/or energy content. There are several other factors besides the nutrient content of foods that make up a healthy dietary pattern and require more attention in FBDGs. Several of these factors are food-based (e.g., phytonutrient content, probiotics, the food matrix, food synergy, processing, and preparation effects) while several are not (e.g., speed and timing of food intake, genomics, the microbiome). However, at present, the key messaging in many FBDGs heavily focuses on the basic nutrient content of foods [14], as these have traditionally been the datasets available in national databases. Only recently are datasets on bioactive food components and processing becoming available, which can be used to better inform future FBDGs of overall diet quality and healthy dietary patterns. This type of additional information can provide a much more comprehensive view on the roles of different foods and food groups in healthy dietary patterns.

## 7. Food Matrix Effects and Food Synergy—Emerging Themes in Food and Nutrition Science

While fruits, vegetables, and dairy foods all provide a unique, and sometimes overlapping spectrum of attributes that benefit human health, these food components do not act in isolation. Rather, the thousands of compounds in each of these foods are chemically and physically bound together to form a food’s distinctive structure, known as its matrix. A food’s matrix is responsible for many of its functional properties since the compounds within the matrix work off each other to influence the food’s flavors and textures, as well as to impact digestion, nutrient release, and absorption. For example, the proteins, lactose, and vitamin D in dairy foods are all able to enhance the absorption of calcium through different mechanisms of action, and the intake of certain dairy proteins can improve the absorption of vitamin B2, vitamin B12, folate, magnesium and zinc [6,38]. These types of interactions can also work across foods, with the compounds in one food able to enhance or inhibit the availability and activity of compounds in another. The vitamin C in fruits and vegetables has been shown to enhance the absorption of non-heme iron from grain-based meals [39], and fatty acids from ASF or PSF can improve the absorption of fat-soluble compounds present in low-fat foods [40]. These types of nutrient-nutrient relationships reveal both complementary and synergistic benefits of co-consuming foods from different food groups, and they also reveal that an approach to nutrition science focused only on isolated nutrients will often miss key interactions and relationships responsible for human health.

Additionally, of importance to a food’s functional benefits, is the degree and type of processing it undergoes [41]. Food processing and preparation techniques can enhance or alter a food’s matrix and overall ability to influence health. For instance, processing methods can enhance the amount and/or antioxidant activity of lycopene present in tomatoes [42], and different strains of probiotic starter cultures can impact the nutritional composition and flavor profiles of yogurt [43]. Food processing is a double-edge sword; certain processes such refining or concentrating ingredients, can improve food safety and shelf life, but these processes may simultaneously destroy a food’s matrix and many of its health-promoting properties. On the other hand, microbial processing, in the form of fermentation tends to have an array of synergistic effects, improving safety and shelf-life, as well as the health-promoting value of a food [41,44].

About one-quarter of global FBDGs recommend limiting “ultraprocessed foods” as part of their guidance [1], but the context is critical here and often overlooked. It should be made clear that the type of processing a food undergoes (e.g., mechanical vs. microbial), as well as the degree of processing (e.g., minimally processed vs. ultraprocessed) are both consequential to that food’s effects on health. An oversimplified approach to categorizing the health or quality of foods, focused solely on processing, will have limited use for population health. It has been suggested that nutrient-density is a similar, yet more informative metric than degree of processing for determining how a food will affect health [45]. Overall, the degree of processing and nutrient-composition, along with the bioactive content and synergistic effects of foods, are all important factors to consider for determining the potential health effects of a food.

## 8. The Convergence of Nutrition and Sustainability

Energy, nutrients, food groups, dietary patterns, and human health have traditionally been the focal points of dietary guidance, but nutrition science is no longer the only driving factor in FBDG recommendations. With the globally recognized need to shift to more sustainable food systems, several FBDGs have begun to incorporate sustainability recommendations into their dietary guidance, with some groups even suggesting that FBDGs be reconceptualized to Sustainable Food Based Dietary Guidelines (SFBDGs) [46,47]. These efforts are a work in progress, as an emerging body of science is allowing for a greater overall understanding of the interconnected nature of our food systems to multiple aspects of sustainability (e.g., health, economic, social, and environmental) [48]. At the same time, many different interpretations of the evidence on healthy and sustainable diets are being communicated to the public, with some of the major health concerns focused on the triple burden of malnutrition (undernutrition, micronutrient deficiencies, obesity) and chronic diseases, and the environmental concerns focused on topics such as climate change, water use, land use, energy use, and biodiversity loss [49].

Lessons from ecology reveal that biological systems tend to thrive when they encompass both diversity and redundancy [50]. This key principle is directly applicable to dietary patterns as well. Dietary patterns that are nutritionally diverse (i.e., inclusive of a balanced variety of nutrient-dense foods) and nutritionally redundant (i.e., contain foods with overlapping nutrient profiles) contribute to more secure and resilient food systems than dietary patterns which are limited and exclusionary of major food groups. Dietary advice which recommends a single, prescriptive diet for all humans, regardless of their geographics, demographics, and preferences, is in direct contradiction to the definition of sustainable diets which aim to be inclusive of all of these considerations [51]. This is why there are roughly one hundred different FBDGs available throughout the world rather than a single mandate.

## 9. The Role of Plant-Source and Animal-Source Foods in Healthy and Sustainable Diets

Media messaging regarding healthy and sustainable diets is often framed in terms of food source; with a common theme being that ‘PSF are good’ and ‘ASF are bad’ for health and sustainability [52,53,54]. Although this is a nice and easy way to classify foods, it is much too simplistic from both health and sustainability perspectives. The ‘PSF = good’ and ‘ASF = bad’ assertion sets up a false dichotomy regarding food choice, equating all foods from one source with each other, when both PSFs and ASFs encompass expansive and diverse taxonomic kingdoms, comprised of thousands of dietary options that can be grown, harvested, processed, prepared, and consumed in a myriad of different ways that impact their health value and sustainability. There are also significant overlaps and critical tradeoffs in the health and sustainability impacts among foods which cut across sustainability domains and cannot be easily or accurately quantified using current metrics or frameworks [55].

Diet, health, and sustainability are all extremely complex and dynamic subjects, they require much more nuance than being distilled down to a ‘good’ or ‘bad’ type of binary decision. The focus could instead be on determining and promoting the most complementary and/or synergistic options from both sources since both PSFs and ASFs can provide dietary options that can be healthy and sustainable. The wording of the UN’s High-Level Task Force on Global Food and Nutrition Security summed this premise up nicely with a statement in their report on food system sustainability stating “*There is no one model of a sustainable foods system, but a set of principles that constitute sustainability*” [56]. These overarching principles are highlighted in multiple Food and Agriculture Organization (FAO) publications, and generally specify that research strategies for healthier and more sustainable diets and food systems should: 1) use a holistic approach considering all sustainability domains—health, environment, social, and economic; 2) take a collaborative approach, working across scientific disciplines, industry sectors, and national borders; 3) improve data collection/sharing, standardize metrics, and depend on evidence-based decision making [56,57,58].

### 9.1. Environmental Sustainability—Impacts, Trade-Offs, and Ecosystem Services

The majority of FBDGs make it clear that consuming a balance of PSFs and ASFs is one of the major tenets of achieving a healthy diet [1]. Yet, it is much less clear if this advice to consume a balance of PSFs and ASFs is also true for achieving an environmentally sustainable dietary pattern. ASFs tend to have higher resource needs than PSFs, and are also generally associated with greater environmental impacts, making higher PSF and lower ASF intake seem ideal for environmental sustainability. However, not all PSFs are created equal, nor are all ASFs. Just as there is a range of nutritional attributes, there is also a range of sustainability impacts for each. For example, the carbon footprint for ASFs can range by more than 5-fold even for the same product (e.g., milk) when produced in different regions or under different production methods and can range by more than 25-fold between different types of ASFs, such as intensively produced milk (1.3 Co2 eq/kg) and certain kinds of meat (34 Co2 eq/kg) [59,60,61]. ASFs with relatively smaller carbon footprints such as milk, eggs, and chicken can have similar carbon footprints to certain PSFs (e.g., rice, rapeseed, and almonds) [60,62]. These types of lower-carbon footprint ASFs along with PSFs can both provide essential and complementary nutrients and bioactive dietary components in a planet-smart manner.

In recent years, a number of publications have posited that significant reductions in animal agriculture and increases in plant-based diets would lead to more environmentally sustainable food systems [60,63,64]. While this may be true for certain ASFs and specific environmental impacts (e.g., carbon footprint), it may not be true for all, as there are a vast number of foods, impacts, and trade-offs to consider. Climate is arguably the most important environmental factor to consider, but a food’s carbon footprint is only one measure of its environmental sustainability. Other impacts of importance include water use, land use, energy use, pesticide and/or fertilizer use, impacts of biodiversity loss, with some of these environmental impacts varying up to 50-fold among similar food products [65]. On the other hand, there are also several positive contributions of foods to the environment such as ecosystem services (i.e., direct and indirect contributions to ecosystem health) that should be equally accounted for when aiming to transition food systems to healthier and more sustainable means [66].

Plant and animal agriculture both utilize environmental resources, and both provide environmental benefits (i.e., ecosystem services) that are largely underappreciated for their effects on human and planetary well-being. Plants help remove carbon dioxide from the atmosphere and are thereby directly involved in climate regulation; they produce the oxygen, food, and habitat in which multiple lifeforms are dependent; they help to recycle nutrients throughout ecosystems; they filter and purify water; and they protect soils from erosion. Plants can also be used for the production of textiles, building materials, fuels, and medicines, which all lessen human dependency on extractive resources and synthetic options for these raw materials. In other words, we are living in a plant-based world, in which humans are dependent on plants for the air we breathe, food we eat, clothes we wear, and houses we live in.

**Figure 1:**
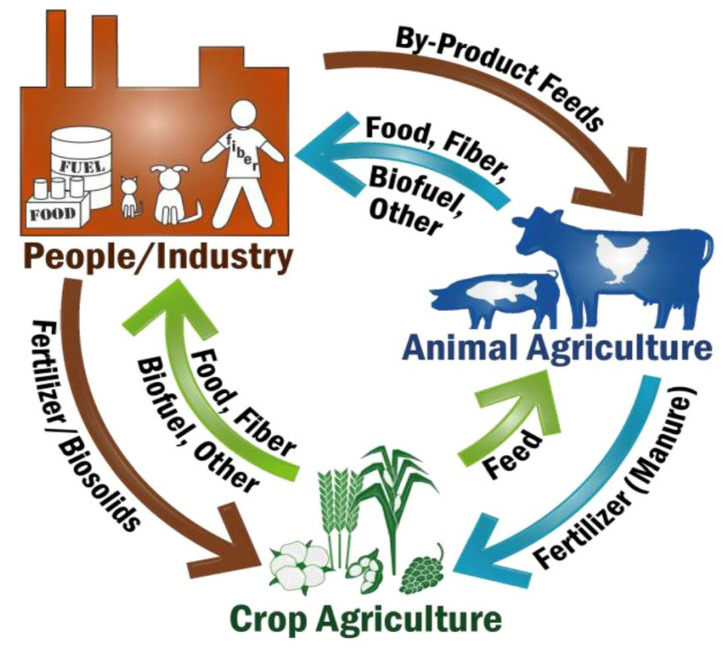
The Symbiotic and Complementary Roles for Plant and Animal Agriculture. Figure reprinted with permission from White et al, 2017 [68] under CC BY-NC-ND license. https://doi.org/10.1073/pnas.1707322114.

Although animal agriculture can be resource intensive and have significant environmental impacts, animal production systems also deliver essential functions for human and planetary well-being. One such service is nutrient recycling and upcycling, in which ruminant animals utilize millions of tons of plant-based byproducts for feed and bedding on an annual basis, most of which are not edible by humans and might otherwise become waste products and CO_2_ producers [67,68]. Furthermore, ruminant livestock which graze on lands that are not suitable for human-edible crop production can upcycle low-nutrient human inedible plants into nutrient-rich ASF, while at the same time producing nutrient-rich manure that can be used as natural composts and fertilizers [69]. Manure-based composts and fertilizers can be used to naturally improve the quality and fertility of the soils in which PSF crops are grown. Both animal and plant byproducts can be used as substrates for biofuels, thus reducing dependency on fossil fuels and lowering agriculture’s overall carbon footprint. All of these ecosystem services show that plant and animal agriculture have a symbiotic and complementary relationship, in that they help each other to stay in balance, and need each other to thrive (Figure 1).

### 9.2. Social and Economic Considerations—The Forgotten Domains of Sustainability

In LMICs, many of the ecosystems services associated with plant and animal agriculture may even be considered more valuable than currency as they provide multiple goods and services that are directly necessary for survival and retain their value in times of economic uncertainty [70,71]. The exact financial values of these types of goods and services largely fall into informal or “hidden” economies making them difficult to quantify. Nonetheless, these provisioning and regulating services provided by both plant and animal agriculture are critically valuable to social, economic, and environmental sustainability [72]. These considerations exemplify the complexity of a holistic approach to sustainability in FBDGs. In addition to the ecosystem services they provide, PSF and ASF are each associated with multiple impacts on social and economic sustainability. Billions of individuals throughout the world depend on agriculture for their livelihoods, with food production forming the backbone of the economy in many developing countries and rural areas [73]. On the consumer side, a large percentage of the global population depends on access to affordable and acceptable forms of PSF and ASF for attaining nourishment [74,75]. A drastic reduction in either PSF or ASF in the food supply would be of huge global health concern and socio-economic concern, since plant and animal agriculture both form the foundations of many societies and economies around the world, and they also provide the calories and complementary nutrients to optimally fuel and nourish them as well [74,76].

Despite all the benefits listed above showing the complementary roles that plant and animal agriculture may provide to health, societies, economies, and the environment, there is still an absolute need for major changes to our global food systems to improve public and planetary health. Simply aiming for more balanced and more sustainable diets will not be sufficient to achieve critical FBDG targets or the Sustainable Development Goals (SDGs). Much more work needs to be conducted to improve the health and sustainability of plant and animal agriculture. Both have room for improvement, and both will need to be improved to be able to nourish a growing population while using fewer natural resources and producing less waste. Among the many suggestions for how to do this, include improving/innovating on research methods, data collection, technology, and collaboration between stakeholders [77]. Efforts and initiatives tackling these issues are currently underway, and many are showing promise for improving the health and sustainability of food systems and/or dietary patterns.

## 10. Moving in the Right Direction with International and Multi-Sector Collaborations

At the global level, FAO and World Health Organization (WHO) have been moving toward a dual focus on both healthy and sustainable dietary patterns. In a joint effort, the FAO and WHO released a set of guiding principles for “Sustainable Healthy Diets”, which aim to incorporate all dimensions of sustainability into dietary guidance [57]. The overarching goals of this guidance is to simultaneously achieve diets that can promote human health and socio-economic wellbeing, while also preserving biodiversity and protecting planetary health. New voluntary guidelines on food systems and nutrition have also been developed by the UN Committee on World Food Security (CFS) which call for the inclusion of multiple dimensions of sustainability, rather than just a singular focus on human health [78].

As the science of sustainability progresses, both plant and animal agricultural sectors are working toward improving production efficiencies and reducing their environmental impacts. In recent years, fruit and vegetable sectors as well as the global livestock and dairy industries have joined forces to form country-specific and international initiatives aimed at reducing environmental impacts [79,80,81,82,83]. The areas of greatest effort for these initiatives are reducing greenhouse gas emissions, water use, energy use, and food waste; while improving soil health, biodiversity, and waste management through the development of improved metrics and the implementation multiple strategic interventions across the globe [84]. These types of collaborations are a natural fit as plant and animal agriculture both complement and depend on each other. They are mutually beneficial facets in the food web, each providing their own set of ecosystem services, and working together through a symbiotic nutrient cycle that benefits soil, plants, animals, and humans.

New science-based resources are also being developed to be used in conjunction with FBDGs, that simultaneously address nutrition and sustainability at national, regional, and global levels. The Food Systems Dashboard is one such tool that aims to transforms data from over 140 food system inputs into publicly available infographics and guidance for improving food policy, programming, and investments [85]. It was developed by a multidisciplinary team of researchers and pulls together data from dozens of major cross-sectoral entities like the United Nations and World Bank. These massive public and private sector collaborations inclusive of diverse perspectives and interests in agriculture, health, and sustainability, further emphasize that future solutions will be aimed at addressing both health and sustainability together, rather than each one individually or in isolation.

## 11. Conclusions

Both PSF and ASF can have roles to play in nourishing a growing global population, and to ensure that food supplies, livelihoods, markets, and traditions throughout the world can flourish. Additionally, both PSF and ASF both have important roles to play in planetary health. The binary argument of plant versus animal is responsible for much contention within the healthy and sustainable diets dialogue, but we proffer that food source is *not* the most useful or informative comparator for assessing healthy and sustainable diets. These issues are much more complex than whether a food comes from plant or animal origin. The practices and places in which foods are produced, processed, transported, packaged, priced, sold, stored, prepared, shared, combined, consumed, and wasted, are all key determinants in how they influence health and sustainability measures. These are all factors that need significantly more research and attention in FBDGs.

There is no denying that food systems and dietary patterns must drastically change to be healthier and more sustainable. Many FBDGs offer a road map as to how to accomplish this, but few countries have been effective in operationalizing them [86]. In addition to the essential steps of improving dietary diversity and variety, a move towards increased consumption of nutrient- and bioactive-rich foods such as fruits, vegetables, and dairy foods in place of lower-nutrient and higher environmental-impact foods is an actionable food-based step toward a solution [54]. These foods can provide complementary combinations of essential vitamins and minerals, healthy fats, fiber, protein, phytonutrients, and probiotics necessary for optimizing human health.

In sum, there is a need to bring much more nuance and context to the discussions surrounding diet, health, and sustainability in FBDGs. There is no single or simple answer as to which dietary pattern is most healthy and sustainable; rather there are many possible patterns, each with their own unique synergies and tradeoffs. To this end, FBDGs should continue to optimize their guidance towards promoting the most essential, complementary, and synergistic dietary options available for both human and planetary health – as well as for improving social and economic outcomes.

## Figures and Tables

**Table 1:** Macronutrients Commonly Associated with Plant-Source and Animal-Source Foods.

	Plant-Source Foods				Animal-Source Foods	
	Fruits: Fresh, Frozen, Canned, Dried	Vegetables: Dark Green, Red & Orange, Roots, Tubers	Grains: Whole, Fortified, Enriched	Protein Foods: Legumes, Nuts, Seeds, Soy Products	Protein Foods: Meat, Eggs, Poultry, Seafood	Dairy: Milk, Yogurt, Kefir, Cheese
**Carbohydrates**						
Fructose	X					
Lactose						X
Polyols	X	X				
Oligosaccharides	X	X	X	X		X
Starch	X Plantain, Banana	X	X	X		
Fiber	X	X	X	X		
**Fats**						
Saturated						
* Short-Chain*						X
* Medium-Chain*	X Coconut, Palm					X
* Long-Chain*					X	X
MUFA	X Olive, Avocado			X Nuts	X	X
**PUFA**						
* Omega-3:* *ALA*			X Flax, Chia	X Walnuts, Soy	X Enriched Eggs	X
* Omega-3:* * DHA/EPA*					X Fish, Beef, Lamb	X Fortified Products
* Omega-6:* * Linolenic Acid*		X Corn	X Flax, Hemp	X Sunflower, Soy	X	
* Omega-6:* * CLA*					X	X
Odd Chain						X
**Proteins**						
Complete				X Soy	X	X
Incomplete	X	X	X	X Legumes, Nuts, Seeds		

Nutrient information sources: (ods.od.nih.gov/factsheets/) [8], (nutritiondata.self.com/tools/nutrient-search) [9]. If only a specific subset of foods within a food group commonly contains the dietary component, examples of these foods are listed in the table.

**Table 2:** Micronutrients Commonly Associated with Plant-Source and Animal-Source Foods.

	Plant-Source Foods				Animal-Source Foods	
	Fruits: Fresh, Frozen, Canned, Dried	Vegetables: Dark Green, Red & Orange, Roots, Tubers	Grains: Whole, Fortified, Enriched	Protein Foods: Legumes, Nuts, Seeds, Soy Products	Protein Foods: Meat, Eggs, Poultry, Seafood	Dairy: Milk, Yogurt, Kefir, Cheese
**Vitamins**						
Pre-Vitamin A			X Fortified Cereals		X	X
Pro-Vitamin A	X	X				
Vitamin B1			X	X	X	
Vitamin B2			X	X	X	X
Vitamin B3			X	X	X	
Vitamin B5			X	X	X	X
Vitamin B6			X	X	X	
Vitamin B7			X	X	X	
Vitamin B9		X	X	X	X	
Vitamin B12					X	X
Vitamin C	X	X	X Fortified Cereals			
Vitamin D		X Mushrooms	X Fortified Cereals	X Fortified Products	X Seafood	X Fortified Products
Vitamin E		X	X	X		
Vitamin K		X		X		
**Minerals**						
Calcium	X Fortified Juice	X Dark Greens	X Fortified Cereals			X
Chromium			X		X	
Copper		X Potatoes, Mushrooms	X	X	X	
Iodine					X Seafood	X
Heme-Iron					X	
Non-Heme Iron	X	X Leafy Greens	X Fortified Cereals	X		
Magnesium	X	X Dark Greens, Potatoes	X	X	X	X
Manganese		X Leafy Greens	X	X	X	
Molybdenum		X Leafy Greens	X	X		X
Phosphorus		X Potatoes	X	X	X	X
Potassium	X	X	X	X	X	X
Selenium			X	X	X	X
Sulfur		X Cruciferous, Allium, Greens	X	X	X	
Zinc			X	X	X	X

Nutrient information sources: (ods.od.nih.gov/factsheets/) [8], (nutritiondata.self.com/tools/nutrient-searchs) [9]. If only a specific subset of foods within a food group commonly contains the dietary component, examples of these foods are listed in the table. In this table, mushrooms are considered a vegetable since they are classified as vegetables in the Dietary Guidelines for Americans [10], although they are technically fungi–and therefore from a whole different biological kingdom than plants or animals.

**Table 3:** Bioactive Food Components Commonly Associated with Plant-Source and Animal-Source Foods.

	Plant-Source Foods				Animal-Source Foods	
	Fruits: Fresh, Frozen, Canned, Dried	Vegetables: Dark Green, Red & Orange, Roots, Tubers	Grains: Whole, Fortified, Enriched	Protein Foods: Legumes, Nuts, Seeds, Soy Products	Protein Foods: Meat, Eggs, Poultry, Seafood	Dairy: Milk, Yogurt, Kefir, Cheese
**Bioactive Food Components**						
Anti-Nutrients						
* * *Lectins*			X	X		
* * *Phytates*			X	X		
* * *Oxalates*	X	X	X	X		
Sterols						
* * *Cholesterol*					X	X
* * *Phytosterols*	X	X	X	X		
Polyphenols						
* * *Flavonoids*	X	X		X Soy		
* * *Phenolic Acids*	X					
* * *Lignans*	X		X	X		
* * *Stilbenes*	X			X Peanuts		
Glucosinolates		X				
Saponins		X		X		
Carnosine					X	
Creatine					X	X
Choline				X	X	X
Taurine					X	X
Nucleic Acids		X		X	X	
Immuno-globulins						X

Bioactives information sources: Slavin et al., [11] and Woodward et al. [12]. If only a specific subset of foods within a food group commonly contains the dietary component, examples of these foods are listed in the table.
